# Improving Older Asian Immigrants Structural Challenges in Accessing Healthcare Services

**DOI:** 10.1093/geroni/igaf122.3794

**Published:** 2025-12-31

**Authors:** Sabita Shrestha, Matthew Sprong

**Affiliations:** Governors State University, University Park, Illinois, United States; Governors State University, University Park, Illinois, United States

## Abstract

Older Asian immigrant population is rapidly growing in the United States. According to the US Census Bureau, this population growth is projected to increase by 6.8 million by 2050. This fast-growing older immigrant population comes with the demand of increased access to improved care and services. However, the system of care and services are often complex and confusing. It is daunting to navigate healthcare services in general. Especially, for older Asian immigrants, the navigation of care and service is even more puzzling who have less familiarity of western system of care. Research has shown that older Asian immigrants often underutilized healthcare services compared to other older population in the United States, thus, presenting poorer health outcomes. One of the reasons that older Asian immigrants underuse healthcare services is due to structural challenges/barriers placed within the healthcare system. Often older Asian immigrants in their later-life restricts opportunities to learn new skills, language, systems, etc., therefore, limiting to understand the complex nature of healthcare services hindering access to needed cares and services that impacts their health and well-being. One way to safeguard older Asian immigrants’ health is to focus on improving structural challenges/barriers within the healthcare system that ultimately results into improved access, care, and services. This poster presentation, based on ecological model, will showcase interventions grounded in health education that aimed to improving older Asian immigrants’ structural challenges/barriers. Additionally, the application of intervention strategies will lead to increased and improved access to healthcare and services for older Asian immigrants.

